# Effects of Horizontal Acceleration on Human Visual Acuity and Stereopsis

**DOI:** 10.3390/ijerph120100910

**Published:** 2015-01-19

**Authors:** Chi-Ting Horng, Yih-Shou Hsieh, Ming-Ling Tsai, Wei-Kang Chang, Tzu-Hung Yang, Chien-Han Yauan, Chih-Hung Wang, Wu-Hsien Kuo, Yi-Chang Wu

**Affiliations:** 1 Medical Education Center, Kaohsiung Armed Forced General Hospital, Kaohsiung City 802, Taiwan; E-Mails: wosihan@gmail.com (C.-T.H.); yingchuan@ms.szmc.edu.tw (T.-H.Y.); 2 Department of Pharmacy, Tajen University, Pintung City 907, Taiwan; 3 Institute of Biochemistry and Biotechnology, Chung Shang Medical University and Chung Shang Medical University Hospital, Taichung City 402, Taiwan; E-Mail: csmcysh@csmu.edu.tw; 4 Department of Ophthalmology, Taipei Buddhist Tzu Chi General Hospital, Taipei City 231, Taiwan; E-Mail: doc30845@yahoo.com.tw; 5 Department of Ophthalmology, Tri-Service General Hospital, Taipei City 100, Taiwan; 6 Medical Affairs Bureau, Ministry of National Defense, Taipei City 104, Taiwan; E-Mails: weiching8277@gmail.com (W.-K.C.); b00401122@ntu.edu.tw (C.-H.W.); westlife7833000@yahoo.com.tw (Y.-C.W.); 7 Department of Ear-Nose-Throat, Kaohsiung Armed Forced General Hospital, Kaohsiung City 802, Taiwan; E-Mail: rebecca7255@yahoo.com.tw; 8 Division of Gastroenterology, Department of Internal Medicine, Tri-Service General Hospital, National Defense Medical Center, Taipei City 100, Taiwan; 9 General Educational Center, Center Taiwan University of Science and Technology, Taichung City 406, Taiwan

**Keywords:** acceleration, Gx, Gy, visual acuity, stereopsis

## Abstract

The effect of horizontal acceleration on human visual acuity and stereopsis is demonstrated in this study. Twenty participants (mean age 22.6 years) were enrolled in the experiment. Acceleration from two different directions was performed at the Taiwan High-Speed Rail Laboratory. Gx and Gy (< and >0.1 g) were produced on an accelerating platform where the subjects stood. The visual acuity and stereopsis of the right eye were measured before and during the acceleration. Acceleration <0.1 g in the X- or Y-axis did not affect dynamic vision and stereopsis. Vision decreased (mean from 0.02 logMAR to 0.25 logMAR) and stereopsis declined significantly (mean from 40 s to 60.2 s of arc) when Gx > 0.1 g. Visual acuity worsened (mean from 0.02 logMAR to 0.19 logMAR) and poor stereopsis was noted (mean from 40 s to 50.2 s of arc) when Gy > 0.1 g. The effect of acceleration from the X-axis on the visual system was higher than that from the Y-axis. During acceleration, most subjects complained of ocular strain when reading. To our knowledge, this study is the first to report the exact levels of visual function loss during Gx and Gy.

## 1. Introduction

**Figure 1:**
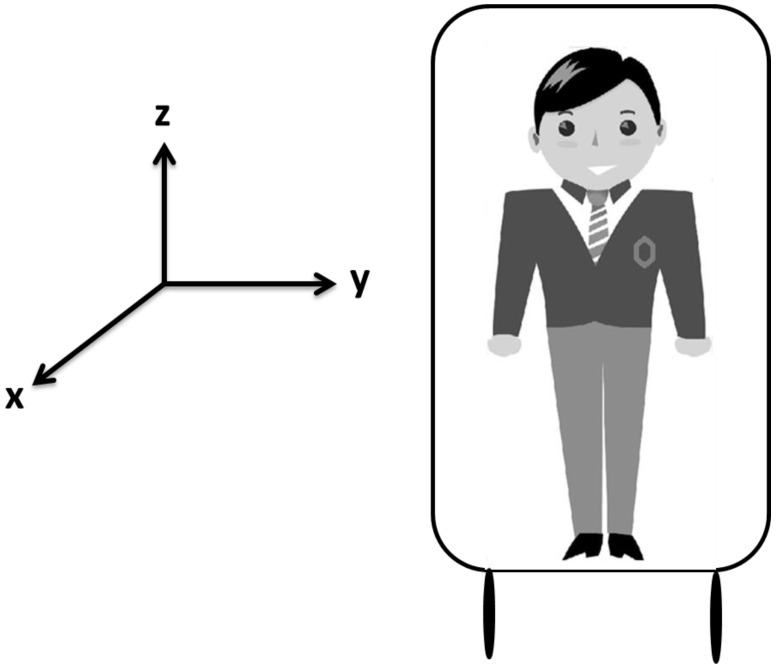
The picture shows three directions (X-, Y-, and Z-axes).

The downward acceleration of gravity from the head to foot (Gz; has been well discussed in the literature (Figure 1). Military aircrews may endure higher Gz (up to 9 g, where g is the abbreviation of the acceleration of gravity = 9.8 m/s^2^) when fighter planes climb rapidly. At emergent acceleration, most blood would not return to the heart and accumulate in the lower limbs, therefore, ischemia and hypoxia of the brain and eyes affect pilots, resulting in a series of visual impairments, such as gray-out, black-out, loss of peripheral vision, and loss of consciousness after encountering a high acceleration [1]. However, the exact vision changes from the horizontal acceleration of X- and Y-axes have been rarely studied. Object recognition critically depends on motion perception, which is associated with the velocity and acceleration of the target. The accelerations from the lateral (left-to-right) direction (acceleration from X-axis, Gx) and anterior-posterior (A-P) direction (acceleration from Y-axis, Gy) are ignored in aerospace medicine. Gx or Gy is not a key issue for aircrews in the sky. Nevertheless, the effect of these two accelerations cannot be neglected on the ground. In 2014, the association between vibration and acceleration in vehicles began to be addressed [2]. Various acceleration mechanisms are seen in daily activities, such as walking, running, driving, travelling, and working in some vehicles (cars, high-speed rails, rapid transit systems, boats, railways, and airplanes, particularly helicopters) [3]. Humans such as sailors, aircrews, and passengers may experience problems, including motion sickness and spatial disorientation, when they are inside moving equipment. Many ground workers also experience vibration and acceleration in their environments and machines, such as construction machineries (bulldozers, forklifts, and cranes), heavy equipment (grinders and jack hammer), and power hand tools [4]. Currently, the effect of horizontal acceleration on visual acuity (VA) and stereopsis remains ambiguous. In this work, we explore the exact changes during Gx and Gy acceleration. 

## 2. Materials and Methods

**Figure 2:**
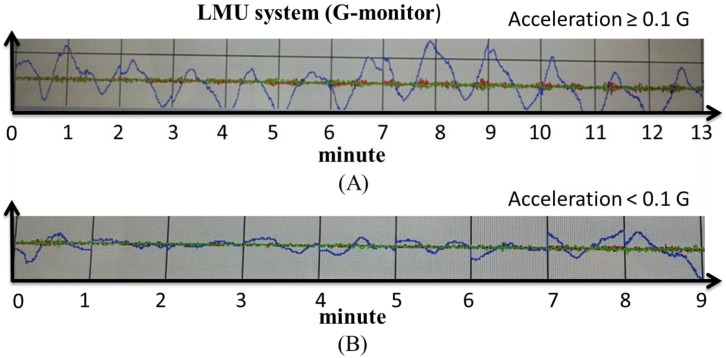
The accelerating speed of the platform was created using a machine, and the acceleration were monitored with an LMU system (G-monitor). Acceleration was approximately or moderately higher than 0.1 G (**A**). Acceleration was less than 0.1 G (**B**).

**Figure 3:**
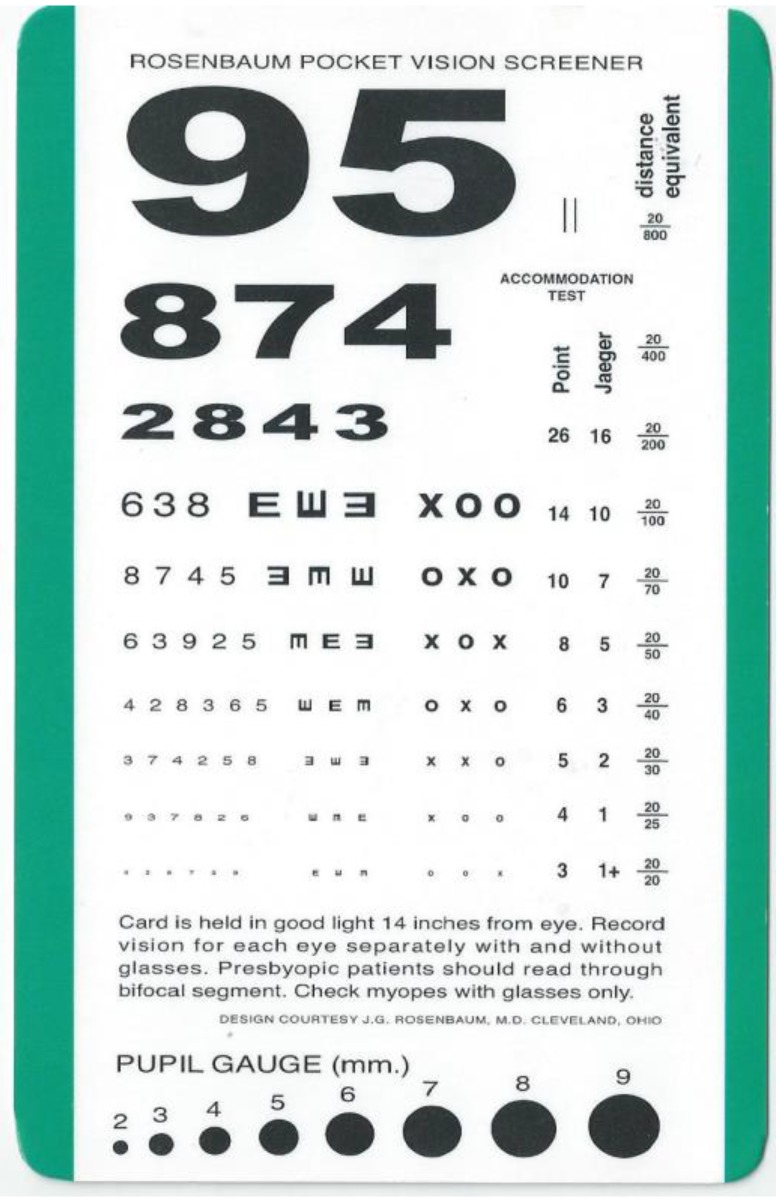
The portable Rosenbaum pocket vision screener is extensively used in clinics to evaluate vision in immobile or sick patients or when the test distance is short.

**Figure 4:**
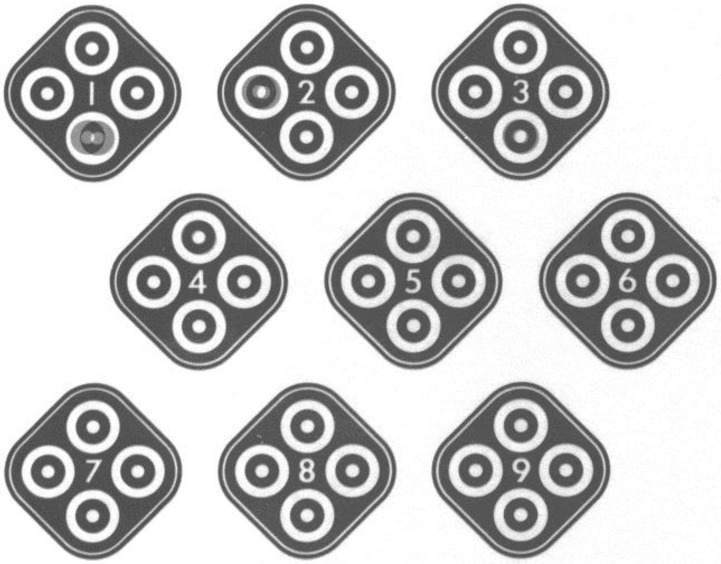
Stereopsis was tested using the picture card of Stereotest-circles (this card contains a total of nine square pictures and only one circle was presented as “forward” to the participants in each square. In this test, all subjects should wear special glasses and determine if the forward phenomenon persisted or not). The unit of stereopsis is shown as second (s) of arc.

Informed consents were obtained from all participants. All experiment protocols were conducted in accordance with the Declaration of Helsinki. Ethical approval was obtained from the Institution Review Board of Kaohsiung Armed Force General Hospital (Kaohsiung, Taiwan). Participants with a history of ocular disorders (except myopia) or systemic diseases, such as hypertension, diabetes, autoimmune diseases, cataract, glaucoma, or uveitis, were excluded from the study. All experiments were performed at the Taiwan High-Speed Rail Laboratory (Kaohsiung City) in May 2014. A total of 20 adult participants, including 15 males and five females, were enrolled in the study. The subjects were aged between 18 and 24 years old (mean age was 22.6 years). Refractive errors were between +1.0 *D* and −3.0 *D* and could be corrected to 20/20 by using glasses (best corrected visual acuity; BCVA). No cranial nerve diseases, such as vestibular problems, were found. Common cold medications and drinking alcohol were prohibited at 3 d before the study. All subjects presented no evident symptoms of upper respiratory tract infection that could affect the vestibular function. Subjects with vestibular diseases frequent complain of unsteady visual sensations and blurred vision during head movements. This phenomenon is termed oscillopsia; hence, these subjects were excluded from our study [5]. All 20 subjects were initially checked on the ground (static VA and static stereopsis). An accelerating platform was subsequently created using a special machine, and the degree of speed was monitored with a local measuring unit (LMU) system. Each participant was asked to stand on the platform and was then subjected to dynamic VA and dynamic stereopsis tests. In this study, we designed two types of acceleration (moderate higher than 0.1 g and lower than 0.1 g) (Figure 2A,B) and two directions (from the X- and Y-axes). VA was determined from the right eye of the 20 subjects. During the moving platform experiment, all participants were asked to cover their left eyes and read the letters on the Rosenbaum pocket vision card by using their right eyes with and without their glasses. The results indicated the VA of the right eye (Figure 3). After completing the VA examination, a participant was subjected to stereopsis test using the Stereotest-Circles (Stereo Optical Co., Inc., Ltd., Chicago, IL, USA) with both eyes (Figure 4) (the subjects should wear special glasses in this experiment). All results were recorded in detail. After the test was completed, the accelerating platform was stopped. Another subject then went up on the stopped platform and underwent similar procedures. 

The Rosenbaum pocket vision screener was used in this study because of its convenience and short distance requirement (about 35 cm). This screener is clinically used to examine the vision of immobile patients in the ICU or people with trauma. VA results can be converted to the equivalent Snellen chart (measure distance is about 6 m) and corresponded to 20/20, 20/25, 20/30, 20/40, 20/50, 20/70, 20/100, 20/200, 20/400, and 20/800 in this study. Static VA represents the vision checked on the ground, whereas dynamic VA denotes the vision measured on the accelerating platform. Finally, the results of VA were transferred to another visual acuity scale (log MAR). The letter size of each line is designated as the logarithm to the base of 10 of a decimal VA; for example, the 20/20 line is equal to 0.00 log MAR 0.00 and the 20/25 line is similar to 0.10 log MAR. The log MAR is used in many studies to represent VA because it can be easily calculated [6]. Log MAR is derived from the logarithm of the minimum angle of resolution. The opto-type size varies in each line by 90%, representing a logarithmic change in the minimum angle resolvable (log MAR) of approximately 0.1 unit [7]. For example, the World Health Organization (WHO) established criteria for low vision using log MAR scale. Low vision is defined as a BCVA higher than 0.5 log MAR, and blindness is represented by BCVA higher than 1.3. The test task can be easily standardized using the log MAR method [8]. Therefore, log MAR became the standard psychophysical method for assessing VA and is considered appropriate for quantitative measurement by many scholars. Moreover, the Stereotest-Circles test is extensively used by many ophthalmologists to evaluate the ability of image fusion, namely, stereopsis. This method has also been used to evaluate changes in visual function during Coriolis illusion in pilots [9]. In the test, the subjects may view the nine square pictures with special glasses in both eyes. Each square picture contains four circles at the upper, lower, left, and right parts. In normal stereopsis, the subject may regard the circular projection as “forward,” which can be measured as 800, 400, 200, 140, 100, 80, 60, 50, and 40 s of arc. Excellent fighter pilots may exhibit 40 s of arc; however, stereopsis of patients with various diseases decreases. In our experiment, the participants evaluated on the ground demonstrated static stereopsis, whereas those in the shaking platform presented dynamic stereopsis. The sensation of the subjects during acceleration while reading the test card was also recorded. All statistical analyses were performed using IBM SPSS Statistics version 21 (IMB Corp., Armonk, NY, USA). Results are expressed as mean ± SD. Paired t-tests were used to compare BCVA with stereopsis in the ground (static results) and acceleration phase (dynamic results). A *p* value lower than 0.05 was considered statistically significant. 

## 3. Results

The BCVA of the right eye in each participant revealed 20/20 in the ground (static VA) with stereopsis reaching 40 s of arc (static stereopsis). Static BCVA and stereopsis remained constant during horizontal acceleration (<0.1 g) in the X and Y axes. Hence, static BCVA and stereopsis were approximately equal to dynamic BCVA and stereopsis in the accelerating environment, respectively (Gx and Gy < 0.1 g). However, these factors significantly changed when horizontal acceleration was higher than 0.1 g. Dynamic BCVA significantly decreased (from 0.02 log MAR to 0.19 log MAR) (*p* < 0.05) when acceleration in the Y direction was higher than 0.1 g (A–P direction). Therefore, dynamic BCVA of 40% (8/20) of the subjects decreased by one letter line (from 20/20 to 20/25 in the Snellen chart). 

Dynamic stereopsis declined (mean from 40 s to 50.2 s of arc) (*p* < 0.05) in 40% (8/20) of all participants (Table 1). Dynamic BCVA significantly decreased (from 0.02 log MAR to 0.25 log MAR) (*p* < 0.05) when the acceleration (Gx > 0.1 g) was accelerated from the lateral direction (X-axis). Dynamic stereopsis also declined (mean from 40 s to 60.2 s of arc) (*p* < 0.05). About 50% (10/20) of the subjects exhibited reduced dynamic stereopsis of 60 s of arc. All subjects complained of different levels of discomfort and ocular strain while reading the letter cards and stereopsis pictures when acceleration was higher than 0.1 g (Gx or Gy). Thus, we supposed that people in accelerating cars, boats, airplanes, and other vehicles (Gx and Gy > 0.1 g) may experience declined vision and stereopsis (one to two letter lines on the X or Y axis with a visual test card). Moreover, 90% of the subjects may possibly feel mild dizziness, headache, ocular strain, and different levels of discomfort during acceleration.

**Table 1:** Results of VA and stereopsis in the ground (static condition) and during acceleration (dynamic condition) from two directions (X- and Y-axes).

Parameters	Gx < 0.1 g	Gx > 0.1 g	Gy < 0.1 g	Gy > 0.1 g
Static BCVA	0.02 ± 0.01	0.02 ± 0.01	0.02 ± 0.01	0.02 ± 0.02
Dynamic BCVA	0.04 ± 0.02	0.25 ± 0.04 *	0.05 ± 0.04	0.19 ± 0.08 *
Static stereopsis	40	40	40	40
Dynamic stereopsis	40	60.2 ± 0.7 *	40	50.2 ± 0.8 *

BCVA: Log MAR; Stereopsis: second (s) of arc; * *p* < 0.05, significant.

## 4. Discussion

Many researchers have investigated the Z-axis, which plays an important role in various airplanes and space shuttles. A high Gz stress may affect aircrews because massive amounts of blood would remain in stasis in the lower part of the bodies and organs, thus inducing many physiological changes, including visual problems. VA is transiently reduced under rapid acceleration (Gz about 5 g) [10]. Peripheral vision loss and total blindness of aircrews may also occur if this condition persists. Moreover, most people experience a similar condition in roller coasters, which generate 4–5 g Gz on the ground. Travellers may experience compression on their seats during emergent acceleration. In addition, shortness of breath, anxiety, and several serious problems may occur. Cases with internal carotid artery or vertebral artery dissection, brain injury, stroke, sub-dural hematoma, acute soft-tissue injury, neurologic complications, and death have further been reported [11,12,13,14,15,16]. Incorrect and improper G force induced by roller coaster rides may cause visual impairment and ocular complications, such as retinal artery occlusion, glaucoma, lens dislocation, intrao cular lens dislocation after cataract surgery, and macular hemorrhage [17,18,19,20]. 

The effect of horizontal acceleration (Gx and Gy) on the visual system (dynamic VA) during flying and in the ground has been rarely discussed. The moving force from 2.2 g to 7.1 g in the Y-axis possibly indicates the tolerance to lateral acceleration [21,22]. Anecdotal evidence has been derived from race car drivers, who require specific strength training regimes to tolerate 4–5 g Gy loading during cornering [23]. The velocities of the images on the retina range from 2 Hz to 4 Hz under normal condition. A rapid mobile object cannot be distinctly differentiated if the target moves faster [24]. Nevertheless, motion is unavoidable in human life, including during head movement and vehicular travel [25]. Dynamic VA is a resolution during the relative motion of messages from opt-types or observers [26]. Motion affects dynamic VA compared with static VA. The decreased levels of dynamic VA correspond to the cube of horizontal or vertical opt-type velocity [27,28,29]. Dynamic VA is affected by several factors, including age, relative motion of the target, and observer’s head [23]. The maximum head velocities are up to 90° per second with predominant frequencies up to 2.7 Hz for yaw (vertical-axis) and 8.2 Hz for pitch (inter-aural axis) during running [30]. Lisberger *et al.* [31] reported similar results, in which natural head movement occurs at frequencies higher than 2.0 Hz in normal young subjects. Head movements degrade the acuity by producing motion, particularly acceleration of the retinal images of stationary objects because of compensatory mechanisms. The relative position of human body and head may influence dynamic VA when humans are running fast or when they are inside moving vehicles (two dimensions, 2D) in the ground or airplanes (three dimensions, 3D) in the sky. Vestibulo-ocular reflex (VOR) specifically functions to stabilize gaze in space when the head moves during rapid acceleration. VOR may play an important role in compensating ocular movements and maintaining stable images on the retina, thus allowing humans to clearly see during rapid walking, turning heads, or looking out of the window in a car [32]. Moreover, neck reflex, vestibule-collic reflex, and vestibulo-spinal reflex may stabilize vision, control posture, perceive head orientation and self-motion in 2D or 3D, and modulate autonomic and limbic activity in response to locomotion and changes in posture. These reflexes are important to drivers in the ground and to military pilots during rapidly accelerated flying [33,34,35]. Patients with vestibular deficits who encounter a condition similar to visual–vestibular interaction insufficiently produce VOR gain, which should be equal to the required values; hence, retinal image motion is generated during head motion, particularly acceleration that affects the body [25]. Many scholars conclude that retinal slip velocity reduces dynamic VA [36]. 

Identifying targets or letters in mobile environment, such as in driving, flying, and some special exercises, requires excellent dynamic VA because of high tactile skill requirement [37,38]. Dynamic VA is also significantly better among student athletes than among their non-athletic peers [39]. Many clinical physicians focus on diagnosing dynamic VA without using magnetic resonance imaging (MRI), electronystagmography, or other complicated instruments. Patients are requested to see a visual chart before and after head movement. Thus, static and dynamic VA can be easily obtained for further evaluation. For example, patients with dizziness, labyrinthitis, head trauma, multiple sclerosis, and optic neuritis can be easily diagnosed through decreased visual acuity during head motion (a minimum of two-line reduction in dynamic VA). Studies also demonstrated a five-line difference between dynamic and static VA (decline in dynamic VA in these diseases) [40,41]. In addition, dynamic VA remarkably declines during head motion in patients with focal peripheral lesions and aminoglycoside toxicity [42]. Therefore, dynamic VA in patients with vestibular deficits may primarily reflect the degree of vestibular loss [43].

Humans can easily see objects in daily activities in ground through VA, but stereopsis is also important. VA is equal to mono-vision, whereas stereopsis may be considered binocular. The relative position may change when the observed subjects or observers move. Objects cannot be clearly visualized during rapid acceleration. Head movements may affect visual acuity and stereopsis because of the incorrect retinal image that reveals poor capacity for macular sensory fusion. Humans obtain information about visual depth from various sources. Observers can remarkably differentiate between the views of both eyes (binocular disparities) and use the differences to perceive depth and 3D object shape; this phenomenon is called “stereopsis.” Stereopsis begins when the binocular neurons in the primary visual cortex (Brodmann’s area 17) match the images from each eye and compute the disparity of pictures [44]. Japanese scholars used functional MRI and determined that the controlling center is located at the parieto-occipital cortex [45]. Stereopsis is an important component of binocular visual function, particularly retinal image fusion in the horizontal direction. Hence, better stereopsis may enable performance of tasks that require accurate depth perception; such tasks include threading and performing a surgery. Stereoacuity may provide information about the ability of humans to utilize stereoscopic information under operational conditions. The five functional topics that may be important for designing stereoscopic display systems include geometry of stereoscopic depth perception, visual persistence, perceptual interaction among stereoscopic stimuli, neurophysiology of stereopsis, and theoretical considerations. Moreover, pilots, physicians (surgeons), air traffic controllers, meteorologists, professional players, astronomers, scientific staffs, and technical staffs require better stereopsis [46]. 

The development of stereopsis (static stereopsis) is associated with human age. The critical period for human binocular visual function begins at approximately 3 months of age, followed by a period of rapid increase and subsequent development at a slower rate [47]. The stereopsis in 5-year-old children is about 100 s of arc, and the ability to create normal retinal image fusion starts at a mean age of 7 years in the children group. Nine to 13-year old students present 40 s of arc, which is similar to normal adults [48]. Static stereopsis decreases after 50 years [49]. Several studies showed that stereopsis may gradually decline each year. Generally, stereopsis is poor among elderly as their age increases [50]. For example, the average stereo-acuity threshold increases with age from 20 s of arc at 10 years old to about 32 s of arc at 85 years old [51]. Poor stereopsis in older groups is primarily caused by cataract formation, as well as neural and neurophysiological changes associated with aging [52]. These diseases include schizophrenia, Alzheimer’s disease, anisometropia, and various types of strabismus [53,54,55,56,57]. In clinics, stereopsis (static) can be used to effectively evaluate the outcomes and prognosis of many ocular surgeries, such as operations of strabismus and cataracts, as well as refractive surgery. Fawcett *et al.* [58] reported that surgical correction of acquired strabismus is associated with the recovery of stereopsis (250 s elevated to 60 s of arc), which improves during the post-operative adaptation period. Stereopsis is also a significant factor that affects the improvement in vision-related quality of life or depressive symptoms after first eye cataract surgery [59]. 

Dynamic stereopsis is common and important in daily life and some exercises. Vision is a critical element in professional sports, such as baseball. Therefore, vision testing can be used to discriminate good and bad performance. Smart vision is important for hitters and pitchers in Major League Baseball (MLB) in the USA. Hofeldt *et al.* [60] found that most hitters in MLB exhibit a “sharp” dynamic stereopsis of about 40 s of arc. Major league players are significantly more accurate in performing stereophotometry than minor league players. Therefore, stereophotometric testing may be a useful index in predicting batting ability. Solomon *et al.* [61] designed a method to test dynamic stereoacuity and detected subtle differences among individuals. The results show segregation between major league hitters and pitchers. These data can be used to predict hitting performance. Thus, the authors strongly suggest that sportsmen should be screened in terms of dynamic VA. In clinics, dynamic stereopsis is also used to evaluate some diseases. For example, a relatively lower dynamic stereopsis is usually found in patients with schizophrenia [62]. 

The concept of acceleration differs from that of vibration (induced by regular or irregular force). However, these two accelerations persist together in our daily activities. Acceleration persists in one direction, whereas vibration occurs back and forth. These accelerations may result in various physiological effects on human bodies. For example, the shaking forces in the central parts (Nantou city in Middle Taiwan) affected by the 1921 earthquake were 1.5 g of the horizontal acceleration (Gx or Gy) and 0.3 g of the vertical acceleration (Gy). Under this condition, humans may feel severe discomfort. Residents in Taiwan experienced a huge disaster in the earthquake in 1999. Moreover, several convenient transportation systems, such as airplanes, trains, ships, and cars, can induce acceleration and whole body vibration (WBV) that may affect pilots and passengers. WBV refers to the transfer of low-frequency vibrations to areas in contact with the body; such areas include seats of the trucks, tractors, buses, taxis, or other vehicles, as well as floors of workplaces. WBV may also refer to vibration exposures in many occupational settings, such as heavy construction, forklift operation, vehicle operation, and farming. 

The occurrence of vibration in human body causes a series of physiological effects, resulting in muscle contraction. This contraction can stimulate muscle spindle, enhance blood circulation, and improve muscle power. Currently, vibration is widely used by many clinical physicians (particularly in the department of rehabilitation) and in the training programs of various athletes to enhance their performance [63,64,65,66,67,68,69]. However, overexposure to vibration can result in some disorders. Occupational vibration can be categorized into segmental and whole body. Segmental vibration is transmitted through hands and arms and can cause specific health effects, such as Raynaud’s syndrome. WBR is transmitted through the body’s supporting surfaces, such as legs when standing and back and buttocks when sitting. Vibration presents a health risk to the psychomotor, physiological, and psychological systems of the body when exposed to these occupational environments. The most known disorder is “motion sickness,” which affects travelers and drivers during flying and driving, as well as when inside a public road transportation [70,71]. Short-term exposure to vibration within the range of 2–20 Hz at 1 m/s^2^ may induce abdomen pain, headache, chest pain, nausea, loss of equilibrium, muscle contraction with decreased performance in precise manipulation tasks, shortness of breath, and influence on speech [72,73]. Moreover, long-term exposure to vibration may cause more serious health problems, particularly with the spine (disc displacement, degenerative spinal changes, lumbar scoliosis, intervertebral disc disease, degenerative disorders of the spine, and herniated discs) [74,75]. Therefore, many researchers speculated that vibration can cause occupational diseases [76,77]. In addition, studies reported that vibration-induced forces, particularly under long-term exposure, can result in blurred vision. WBR can induce detrimental effects on vision as demonstrated by Grether *et al.* [78]. In their study, broad-band vibration with frequencies higher than 20 Hz at 6 m/s^2^ may cause eye disorders. A constant vibration exposure (frequency 3, 4.4, or 8 Hz; 1.5 m/s^2^) may also result in ocular pain and other problems [79]. Seidel *et al.* [80] supposed that prolonged exposure to WBR may affect eye strain. The effect on vision depends on time and various types of accelerations. The first 10 min generates the most pronounced effects.

The effects of Gx and Gy on vision play an important role in rail-mounted vehicles on the ground; however, this finding has not been further evaluated, particularly in high-speed rail and metro-rapid transit systems, such as Taiwan high-speed rail, Taipei MRT, and Kaohsiung MRT in Taiwan. The VDI guideline 2057 and the International Standard (ISO) 2631 provide indications regarding vibration duration and acceleration exposure per day in the form of curves; such indications are reasonably assumed not to endanger the health of the operators. The intensity of the accelerations provides a good basis to assess whether a specific stress represents a health hazard for the spinal column. The current international standard for WBV (ISO 2631/1-1985) is based on the data available from practical experience and laboratory experiments under short-term exposure. Exposure criteria, including possible source of errors in the public transport systems, satisfy the requirements of the ISO 2631-1; thus, improving the passengers comfort (Table 2). However, accelerations under special conditions may persecute travelers. For example, the Gy acceleration of Taiwan high-speed rail is 0.01 g under normal conditions. People feel good when carriages move in high speed. The Gy acceleration is between 0.03 and 0.06 g during acceleration and deceleration (peak to peak). During this time, passengers may experience mild discomfort without vision loss. However, dynamic VA may slightly decrease (on line letters) and blurred vision may develop when the Gy acceleration is higher than 0.1 g as demonstrated in the present study. In the Taipei and Kaohsiung MRT, the sitting position of passengers and the moving direction is vertical. Hence, the Gx acceleration is predominant in travelers in metro-rapid transit system. The peak of accelerating and decelerating the G acceleration is approximately 0.12 g. The distance from one station to the next station (in Taipei and Kaohsiung City) is also relatively short. During repeated fast moving and stopping of the carriages, the Gx acceleration may decrease the human vision (approximately two lines). Dynamic stereopsis also decreases, and travelers may feel headache and ocular strain as they play smart-phones, iPads, and portable computers for a long time. In the present study, approximately 90% of people may feel ocular strain and dizziness while reading. 

**Table 2:** Effects of vibration on people as defined by the International Standard Organization (ISO). These data were obtained from ISO 2631-1 (1997) as supported and adopted by the Taiwan High-Speed Rail to limit the vibration and acceleration for safety aspects. The health, comfort, perception, and motion of passengers are also considered.

Acceleration	Body of Sensation
<0.03 g	No sensation
0.03–0.07 g	Very mild discomfort
0.07–0.1 g	Mild discomfort
0.1–0.16 g	Moderate discomfort
0.16–0.25 g	Severe discomfort
>0.25 g	Very severe discomfort

Few studies reported visual changes during high-speed or accelerating phase from vehicles. Some studies revealed that the acceleration of cars, buses, and trunks ranges from 0.02 g to 0.09 g in the ground. Mechanical acceleration reaches 0.15 g when vehicles wait for the traffic light signs in the streets [81]. Traffic jams usually occur in large cities (Kaohsiung and Taipei in Taiwan), and repeated speeding up, slowing down, and prolonged waiting may increase the incidence of Gx (or Gy) on humans. A majority (94%) of the accelerations measured in Chen’s study [82] range from 0.02 g to 0.05 g (slightly higher than high-speed rail and metro-rapid transit system in urban taxies in Taipei city). However, prolonged exposure, accumulated effects in traffic jams, and relatively high levels of acceleration cannot be ignored. Thus, we asserted that decreased vision and stereopsis under short-term exposure may aggravate vision loss for a long time. 

Previous methods used to create dynamic models include sitting in a swing rotator in the inter-aural axis [24], computerized dynamic VA test (120°/s) [43], rotator chair [37], passive head movement by physician (e.g., the Hallpike maneuver) [32,41], and active “smooth” (without abrupt changes in velocity or acceleration) head movement or self-generated head movement (0.5 to 3 cycles/s) [42]. However, the created accelerations from these studies are rough and cannot be well-evaluated for calculation; hence, researchers cannot effectively and correctly obtain statistical results. In addition, the velocities are inconsistent in these experiments. In the present study, a special machine (G-inducer, Taiwan) with a platform combined with an LUM system (G-acceleration monitor) was used. To our knowledge, our design is the first that can be used to explore dynamic VA and stereopsis with regard to the acceleration from X and Y axes. Dynamic VA and stereopsis were affected by Gx and Gy. Visual function declined when the G acceleration was higher than 0.1 g in these directions (X- or Y-axis). Convenient instruments, including portable Rosenbaum pocket vision screener and picture cards of Stereotest-circles, were also used because the measured distance on the platform was relatively short. However, our results can be considered reliable because such instruments are extensively used in clinics. 

## 5. Conclusions

The NASA has started to consider the effects of acceleration on reading and visual performance in outer space duties [83,84]. Nevertheless, the secret aspects of this research conducted in the USA have not been reported. To our knowledge, this study is the first to report about the exact levels of visual function loss during Gx and Gy. Our experimental results showed that acceleration <0.1 g in the X- or Y-axis did not affect dynamic vision and stereopsis. Vision decreased (mean from 0.02 logMAR to 0.25 logMAR) and stereopsis significantly declined (mean from 40 to 60.2 s of arc) when Gx was higher than 0.1 g. Visual acuity worsened (mean from 0.02 logMAR to 0.19 logMAR) and poor stereopsis was noted (mean from 40 s to 50.2 s of arc) when Gy was higher than 0.1 g. The effect of acceleration from the X axis on the visual system was higher than that from the Y axis. We also reported that ocular strain and dizziness occur while reading in a rapid moving status. Thus, travelers should not read books or newspapers or play smartphones, iPads, and portable computers in high-speed transportation system to protect their eyes. 
